# Effect of Crosslinking Degree on Sulfonated Poly(aryl ether nitrile)s As Candidates for Proton Exchange Membranes

**DOI:** 10.3390/polym11060964

**Published:** 2019-06-03

**Authors:** Penglun Zheng, Quanyi Liu, Zekun Li, Donghui Wang, Xiaobo Liu

**Affiliations:** 1 College of Civil Aviation Safety Engineering, Civil Aviation Flight University of China, Guanghan 618307, China; 18482179228@163.com (P.Z.); LIzekun96@163.com (Z.L.); cafucwdh@163.com (D.W.); 2 Research Branch of Advanced Functional Materials, School of Materials and Energy, University of Electronic Science and Technology of China, Chengdu 610054, China

**Keywords:** interfacial adhesion, sulfonated poly(aryl ether nitrile)s, proton conductivity, methanol permeability

## Abstract

In order to investigate the effect of crosslinking degree on the water uptake, swelling ratio, and methanol permeability of sulfonated poly(aryl ether nitrile)s (SPENs), the molar content of sulfonated group in bisphenol monomer is fixed at 60% in this work. The properties of sulfonated poly (aryl ether nitrile) with different crosslinking degrees are studied by changing the content of propenyl group in sulfonated poly (aryl ether nitrile)s. The cross-linking reaction of the propenyl groups in the SPENs is cured at 230 °C. All the results show that this method is an effective way to improve the water uptake, swelling ratio, and methanol permeability to meet the application requirements of the SPENs membranes as proton exchange membranes in fuel cells.

## 1. Introduction

Interest has recently increased in direct methanol fuel cells (DMFCs), which directly convert chemical fuels to electricity with high efficiency by utilizing methanol as the fuel [1,2,3,4,5]. DMFCs possess a lot of excellent virtues, such as high energy efficiency, easy fuel storage and usage, and simplicity, which are suitable for applications including public transportation and portable electronics [6,7,8,9,10]. Proton exchange membranes (PEMs), the core part of DMFCs, should exhibit high proton conduction and thus endow fuel cell with high energy conversion efficiency. The most commonly used PEMs materials, Nafion produced by DuPont, possess long-term performance, outstanding chemical stability and excellent proton conductivity [11,12,13,14,15,16]. However, some notable shortcomings, such as low fuel barrier property, high production cost, performance degradation at high temperatures, limit their widespread industrial applications. To overcome these issues, numerous sulfonated aromatic hydrocarbon polymers have been developed as alternative PEMs. These sulfonated aromatic hydrocarbon polymers include sulfonated poly (arylene ether ketone)s [17,18], sulfonated poly(benzimidazoles) [19,20,21], sulfonated poly (arylene ether sulfone)s [22,23,24], sulfonated poly(aryl ether nitrile)s [25,26,27] and sulfonated polyimides [28,29,30].

Among all the sulfonated polyaryl ethers, sulfonated polyaryl ether nitriles possess great potential as proton exchange membranes instead of Nafion membranes for DMFCs [31,32,33,34]. The strong polar nitrile group in SPEN can promote the adhesion of polymer to many substrates through interactions with other polar chemical groups, thus facilitating the adhesion of catalyst to PEM [35,36]. Meanwhile, the presence of side nitrile group can provide potential active sites for polymer crosslinking or further functionalization or exhibit other special properties, such as reducing water uptake and swelling rate without significantly reducing the conductivity of proton exchange membranes [37]. Kim et al. [38] synthesized a series of sulfonated polyaryl ether nitriles (m-SPAEENs) with the same sulfonic acid content by controlling the ratio of binary phenol monomer. Their water uptake and proton conductivity were compared with those of BPSHs and Nafion. The results showed that sulfonated poly (aryl ether nitrile) has better dimensional stability than sulfonated poly (aryl ether sulfone) at similar ionic concentration and higher proton conductivity at approximate water absorption. The reason is that the dipole interaction of nitrile group can effectively inhibit the swelling of m-SPAEENs. Furthermore, the hydrogen bond exists between the sulfonic acid group and the nitrile group through the bridging action of the water molecule, which leads to the increasing aggregation of the sulfonic acid group in the film and thus higher proton conductivity. Kim et al. [39] also confirmed that the ratio of proton conductivity to water absorption of sulfonated poly (aryl ether nitrile) is higher than that of sulfonated poly (aryl ether sulfone) within a certain IEC range. This is mainly due to the strong dipole interaction between the molecular chains of copolymers containing the nitrile group, which limits the swelling in water. Yan et al. [36] synthesized poly (aryl ether ketone nitrile) copolymer (SPPEKN) containing sulfonic group by nucleophilic polycondensation reaction. The thermal stability, water absorption, and dimensional stability of SPPEKN membranes were investigated and compared with sulfonated poly (aryl ether ketone) (SPPEK). SPPEKN copolymers absorb less water and swell less than similar polymers without nitrile group under the same conditions. The presence of strong polar nitrile group significantly enhanced the intramolecular/intermolecular interaction and increased the toughness of the hydrophobic network. As a result, the free volume of water adsorption in SPPEKN membranes was limited, thus reducing the water absorption, and improving the dimensional stability of SPPEKN membranes. A series of sulfonated polyamides containing nitrile group were prepared by Wang et al. [40]. Although their IEC values are very high, the swelling rate of all the membranes is less than 17%, lower than that of some other sulfonated polymers and Nafion 117 with the same IEC values at 80 °C. This is mainly ascribed to the strong dipole interaction between the copolymer chains containing the nitrile group, which can make the film physically cross-linked, thus having good water retention and dimensional stability. In summary, the introduction of nitrile group can improve the comprehensive properties of sulfonated polymers. Therefore, compared with other polyaromatic ethers, sulfonated polyaryl ether nitriles are a kind of polymers with high mechanical properties, high proton conductivity, and excellent dimensional stability. More importantly, the molecular design of SPENs is controllable.

It has been proved to be a powerful and simple method to improve the dimensional and thermal stabilities by cross-linking [41,42,43,44,45,46,47,48]. In our previous work, we have synthesized sulfonated poly(aryl ether nitrile)s with a fixed degree of propenyl group crosslinking [49]. The results showed that sulfonated poly (aryl ether nitrile) with the side chain propenyl group cross-linked possesses the best performance when the content of sulfonated group reaches 60%. In this work, novel sulfonated poly(aryl ether nitrile)s with different degrees of propenyl group crosslinking are synthesized. Herein, the molar content of sulfonated group in bisphenol monomer is fixed at 60%. The properties of sulfonated poly (aryl ether nitrile) with different crosslinking degree can be studied by changing the content of the propenyl group in sulfonated poly (aryl ether nitrile)s. The properties of the cross-linked SPENs membranes were evaluated in order to research the cross-linking mechanism more effectively. The effects of the crosslinking degree on membrane properties such as dimensional stability, water uptake, methanol permeability, proton conductivity, and selectivity have also been investigated.

## 2. Experimental

### 2.1. Materials

Hydroquinone sulfonic acid potassium salt (SHQ), 2,6-Difluorobenzonitrile (DFBN) and Disallyl bisphenol A (DBA, analytical reagent (AR) grade) was obtained from Yantai Hengnuo Chemical Technology Co., Ltd (Shandong, China). Sodium chloride (NaCl, AR) *N*,*N*-dimethylacetamide (DMAc, AR), sodium hydroxide (NaOH, AR), acetone (AR), NMP (AR), and K_2_CO_3_ (AR) were supplied by KeLong (Chengdu, China). All the materials were used without further purification.

### 2.2. SPEN Synthesis

**Scheme 1:**
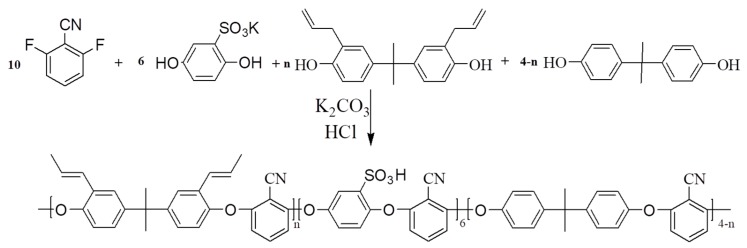
Synthetic route to prepare the crosslinkable sulfonated poly(aryl ether nitrile)s.

Five kinds of sulfonated poly (aryl ether nitriles) with different proportion of propenyl groups were prepared by changing the molar fraction of allyl bisphenol A and fixing the molar fraction of SHQ at 60%. The raw materials for synthesis were shown in Table 1. Typical synthetic routes are were shown in Scheme 1. The typical synthesis route of SPEN-60-DBA-30 was as follows: SHQ (13.68 g, 0.06 mol), Allyl bisphenol A (18.5 g, 0.03 mol), toluene (25 mL), Bisphenol A (2.28 g, 0.01 mol), NMP (65 mL), K_2_CO_3_ (22 g) and DFBN (13.911 g, 0.1 mol) were added to the reaction vessel with a condenser, a nitrogen inlet, a mechanical stirrer, a thermometer and a Dean-Stark trap. The reaction system was firstly heated up to 140 °C and reflexed for 2 h, and then the temperature was slowly raised to 165 °C and polymerized at this temperature about 10 h. Ultimately, the product was precipitated in ethanol, and continuously purified and washed with deionized water and ethanol and dried under vacuum at 100 °C for 24 h.

### 2.3. Characterization 

FTIR spectra were performed by Shimadzu FTIR8400S Fourier Transform Infrared spectrometer (Kyoto, Japan) between 4000 and 400 cm^−1^ in air. Differential scanning calorimetric (DSC) analysis was recorded with TA Instruments DSC-Q100 at a heating rate of 10 °C/min. The thermal degradation (TGA) processes of the membranes were analyzed on a TA Instruments Q50 with a heating rate of 20 °C/min under nitrogen with a purge of 40 mL/min. About 6–10 mg copolymers of the membranes were heated to 160 °C and kept at this temperature for 5 min to remove the residual water and solvent. They were then reheated from room temperature to 600 °C with a heating rate of 20 °C·min^−1^. The mechanical test was conducted on a SANS CMT6104 series desktop electromechanical universal testing machine (Shenzhen, China) at a strain speed of 5 mm·min^−1^ at room temperature; the reported values for each sample were calculated as the average of five specimens.

### 2.4. Ion Exchange Capacity (IEC)

Weight-based IEC (IEC_w_) was determined by the titration method. All the membranes were immersed in 2.0 M NaCl solution for 48 h to liberate the H^+^ ions. Then the H^+^ ions were titrated with 0.01 M NaOH solution using phenolphthalein as an indicator. The titrated IEC was calculated from the following formula:

(1) $$\mathrm{IEC}\;(\mathrm{mmol}/g)=\frac{V_{\mathrm{NaOH}}{\times M}_{\mathrm{NaOH}}}{W_{\mathrm{dry}}},$$

where M_NaOH_ (mol/L) and V_NaOH_ (L) are concentration and volume of NaOH solution, respectively and W_dry_ (g) is the mass of membrane.

### 2.5. Methanol Permeability and Proton Conductivity

The methanol permeability of samples was tested by using a diffusion cell. The cell was consisted of two diffusion cells, each with a volume of about 20 mL, and was separated by a tested membrane. The membranes were hydrated in deionized water for 24 h. Initially, 10 M methanol solution (20 mL) was added in one side of the diffusion cell (cell A) and ultrapure water (20 mL) was added in the other side (cell B). The concentration of the methanol in the water diffusion cell was tested by using a HIMADZU GC-8A chromatograph (Osaka, Japan). The methanol diffusion coefficient was calculated by the following formula:

(2) $$C_{B}(t)=\frac{A}{V_{B}}\frac{\mathrm{DK}}{L}C_{A}(t-t_{0}),$$

where L, A, and V_B_ are the thickness of the membrane, the effective area, and the volume of receptor reservoir, respectively. C_A_ and C_B_ are the methanol concentration in the donor and receptor reservoirs, respectively. DK (in cm^2^·s^−1^) denotes the methanol permeability.

The proton conductivity of the membranes was tested from 10^−1^ Hz to 10^6^ Hz. The membrane samples were hydrated in deionized water at different temperatures for 24 h. The membranes were infibulated between two pairs of stainless-steel electrodes. Conductivity measurements under fully hydrated conditions were carried out with the stainless-steel electrodes immersed in deionized water. The proton conductivity (σ) was calculated by the following formula:

(3) $$\sigma =\frac{L}{\mathrm{SR}}$$

where σ is the proton conductivity (S/cm), L is the distance between the electrodes (cm), R is the impedance of the membrane (Ω), and S is the surface area (cm^2^). Each sample was measured three times to ensure data reproducibility.

### 2.6. Water Uptake and Swelling Ratio

Water uptake was determined by the weight differences that measured between the full-dried and full-hydrated membranes. All membrane samples were immersed in deionized water at different temperatures for 24 h to ensure that the membranes were saturated with water. Subsequently, the water was wiped from the surface of the membranes quickly with blotting paper, and then the weight of wet membranes was measured. The swelling ratio of the membrane sample was determined by immersing it in deionized water at different temperatures for 24 h and measuring the change in length before and after the swelling. The water uptake and swelling ratio of the membranes were calculated using the following equation:

(4) $$\mathrm{Water}\;\mathrm{uptake}=\frac{W_{\mathrm{wet}}-W_{\mathrm{dry}}}{W_{\mathrm{dry}}}\times 100\%,$$

(5) $$\mathrm{Swelling}\;\mathrm{ratio}=\frac{L_{\mathrm{wet}}-L_{\mathrm{dry}}}{L_{\mathrm{dry}}}\times 100\%$$

where W_wet_ and W_dry_ are the mass of wet and dry membrane samples, respectively, L_wet_ and L_dry_ are the length of wet and dry samples, respectively.

## 3. Results and Discussion

### 3.1. Characterization of SPEN and CSPEN

The intrinsic viscosities of SPEN-60-DBA-0, SPEN-60-DBA-10, SPEN-60-DBA-20, SPEN-60-DBA-30, SPEN-60-DBA-40 polymers with different content of allyl bisphenol A were measured by ubbelohde viscometer in a constant temperature water bath kept at 30 °C. The intrinsic viscosities of SPEN-60-DBA-0, SPEN-60-DBA-10, SPEN-60-DBA-20, SPEN-60-DBA-30, SPEN-60-DBA-40 polymers were 1.3, 1.26, 1.23, 1.2, and 1.15 dL·g^−1^, respectively. The higher intrinsic viscosity value indicates that all the sulfonated poly (aryl ether nitrile) products have relatively high molecular weight. The decreasing trend of intrinsic viscosity can be attributed to the lower activity of allyl bisphenol A. Polymerization becomes difficult with the increasing content of allyl bisphenol A.

**Figure 1:**
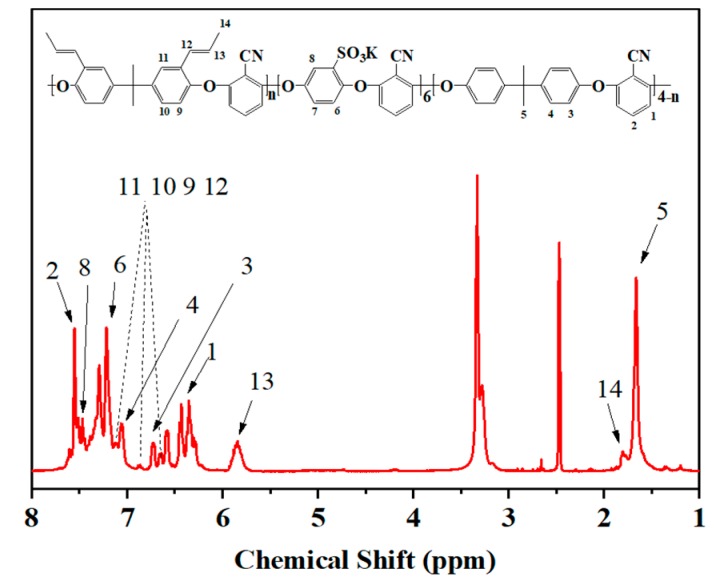
Nuclear magnetic resonance (NMR) spectrogram of SPEN-60-DBA-40.

The structure of sulfonated poly (aryl ether nitrile) polymers with propenyl groups in side chains was verified by nuclear magnetic resonance (NMR). By comparing the ^1^H NMR spectra shown in Figure 1 with the molecular structure of polymers, it can be inferred that the proton singlet at δ5.84 ppm and δ1.81 ppm correspond to the characteristic absorption signals of H_13_ and H_14_ on the acrylic functional group of sulfonated poly(aryl ether nitrile) polymer structure. The proton singlet at δ6.62 ppm, δ6.86 ppm and δ7.12 ppm correspond to the characteristic absorption signals of H_9_, H_10_ and H_11_ on the benzene ring with propylene group of sulfonated poly(aryl ether nitrile) polymer, respectively. The protons signals at δ7.21 ppm and δ7.45 ppm are generated by H_6_ and H_8_ on the benzene ring connecting the sulfonic group, respectively. The protons signals at δ1.66 ppm are the characteristic absorption signal of H_5_ on –CH_3_ on the structure of sulfonated poly (aryl ether nitrile) polymer.

**Figure 2:**
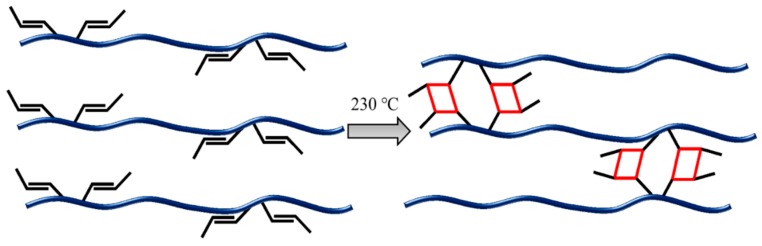
Schematic of the crosslinked polymer chains of sulfonated poly (aryl ether nitrile).

The crosslinking reactions of the propenyl group in sulfonated poly (aryl ether nitrile) polymers can occur when cured at 230 °C, as shown in previous studies [49]. Some propenyl group in sulfonated poly (aryl ether nitrile) molecular chains are opened to form carbon free radicals at high temperature, which continue to attack the propenyl groups in other chains of sulfonated poly (aryl ether nitrile) to form more free radicals. Finally, the carbon free radicals on the sulfonated poly (aryl ether nitrile) chain react with each other to form carbon-carbon single bonds and gradually connect to form cross-linking networks. The specific reaction mechanism is shown in Figure 2. Therefore, the sulfonated poly (aryl ether nitrile) membranes were placed in a vacuum oven at 230 °C for thermal cross-linking treatment. In order to improve the degree of cross-linking of propenyl groups, the treatment time was prolonged to 4 h.

**Figure 3:**
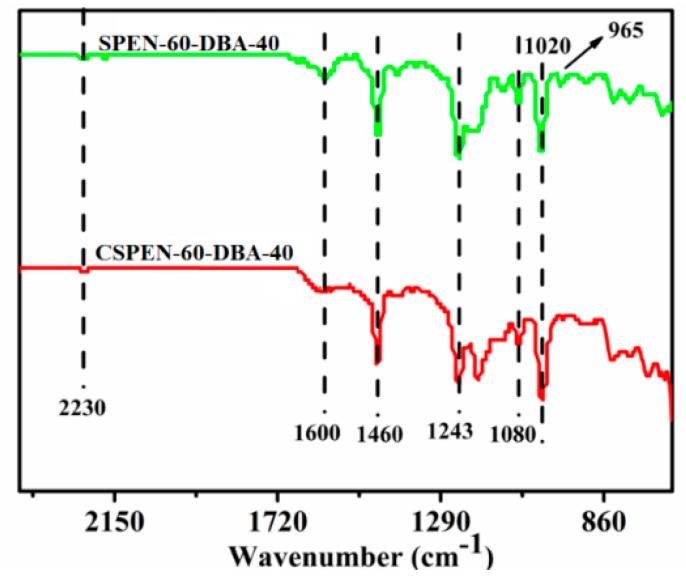
The FT-IR spectra of SPEN-60-DBA-40 and CSEN-60-DBA-40 membranes.

Figure 3 shows the FT-IR spectra of SPEN-60-DBA-40 and CSEN-60-DBA-40 membranes. The absorption bands at 1460, 1600, 1243, and 2230 cm^−1^ all belong to the unique absorption bands of PEN. These characteristic absorption bands can be detected in the FT-IR spectra of SPEN-60-DBA-40 and CSEN-60-DBA-40, which indicates that the sulfonated poly (aryl ether nitrile) has been successfully synthesized. The absorption bands at 1020 and 1080 cm^−1^ can be attributed to the symmetrical and asymmetrical stretching vibration of the sulfonic group (SO^3−^), which indicates that the SO^3−^ has been successfully introduced into the sulfonated poly(aryl ether nitrile). Furthermore, it is worth noting that the stretching vibration absorption bands of the SO^3−^ in CSEN-60-DBA-40 have not been significantly weakened by cross-linking heat treatment, which indicates no decomposition during high temperature crosslinking. Most importantly, it can be clearly found that C=C is located at the absorption bands of 965 cm^−1^ in the infrared spectra of SPEN-60-DBA-40, revealing that C=C has been successfully introduced into sulfonated poly(aryl ether nitrile) [50]. At the same time, when the SPEN-60-DBA-40 was cured at 230 °C, the intensity of the absorption bands at 965 cm^−1^ decreases significantly after crosslinking, which is consistent with the expected results. 

### 3.2. The Gel Content of Sulfonated Poly(aryl ether nitrile)

The gel content of each cross-linked sample was tested by soxhlet extraction. The gel content of sulfonated poly (aryl ether nitrile) with different propenyl group content is shown in Table 2. The gel contents of CSPEN-60-DBA-0, CSPEN-60-DBA-10, CSPEN-60-DBA-20, CSPEN-60-DBA-30, and CSPEN-60-DBA-40 are 0%, 14%, 26%, 43%, and 58%, respectively. The gel content of CSPEN-60-DBA-x is proportional to the amount of propenyl group. 

### 3.3. Thermal Properties

**Figure 4:**
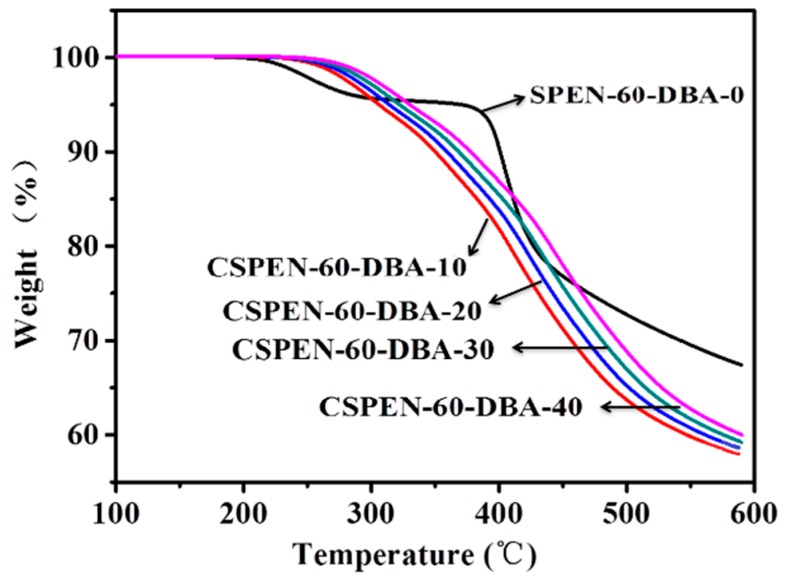
The thermal degradation (TGA) curve of CSPEN-60-DBA-x.

The TGA curve of CSPEN-60-DBA-x is shown in Figure 4. It is clear that all the membranes demonstrate two-step degradation behavior. The first-stage decomposition happens approximately at 245 °C, which is likely caused by the decomposition and shedding of the sulfonic acid group. The second-stage decomposition platform is located at above 400 °C, corresponding to the decomposition of the main chain of sulfonated poly (aryl ether nitrile). In addition, the thermal stability of polymers increases gradually with the increasing content of propenyl group. It is supposed to benefit from the cross-linked networks which can improve the entanglement of polymer chains and inhibit the movement and degradation of the sulfonic acid group. All the membranes show excellent thermal stability before 245 °C, which is higher than that of Nafion 117. Therefore, the sulfonated poly (aryl ether nitrile) membranes with different propenyl group contents exhibit excellent thermal stability, which meets the application requirements of the sulfonated poly (aryl ether nitrile) membranes as PEM materials in fuel cells.

### 3.4. Mechanical Properties

**Figure 5:**
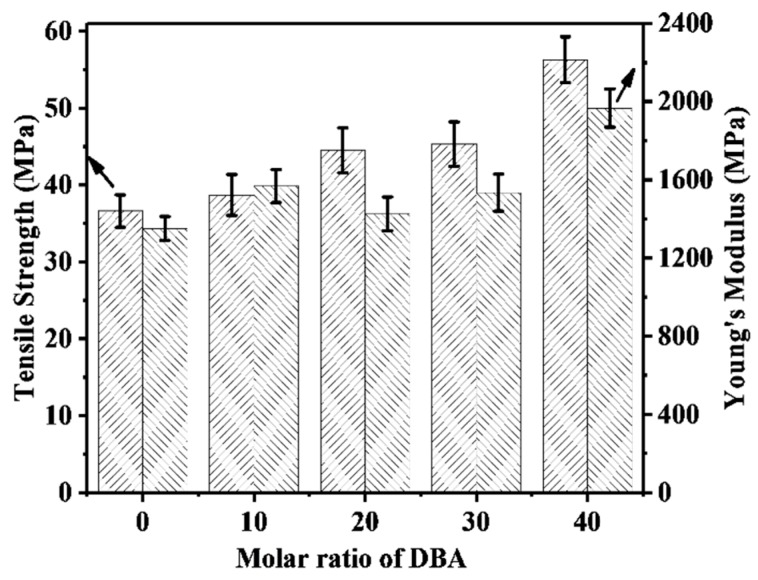
The mechanical properties of CSPEN-60-DBA-x.

The mechanical properties of PEMs are one of the key descriptors to certify if PEMs can be used for long-term operation in fuel cells. The room-temperature tensile strength and Young’s modulus of the membranes are listed in Figure 5. It can be seen from Figure 5 that the tensile strength of CSPEN-60-DBA-x membranes range from 36.6 MPa–56.3 MPa and increase gradually with the increasing contents of propenyl group. The results indicate that cross-linking reaction can greatly improve the tensile strength of the membranes which can induce polymer to form cross-linking structures. The crosslinking networks of CSPEN-60-DBA-x membranes become denser and the entanglement between molecular chains becomes tighter with the increase of propenyl group contents, which leads to the limited movement of molecular chains. It should be noted that the obtained cross-linked membranes exhibit much larger tensile strength than the commercial Nafion 117 membrane (10 MPa). In this aspect, the cross-linking methods can widen their potential application in many special engineering areas.

### 3.5. Ion Exchange Capacity, Water Uptake and Swelling Ratio

**Figure 6:**
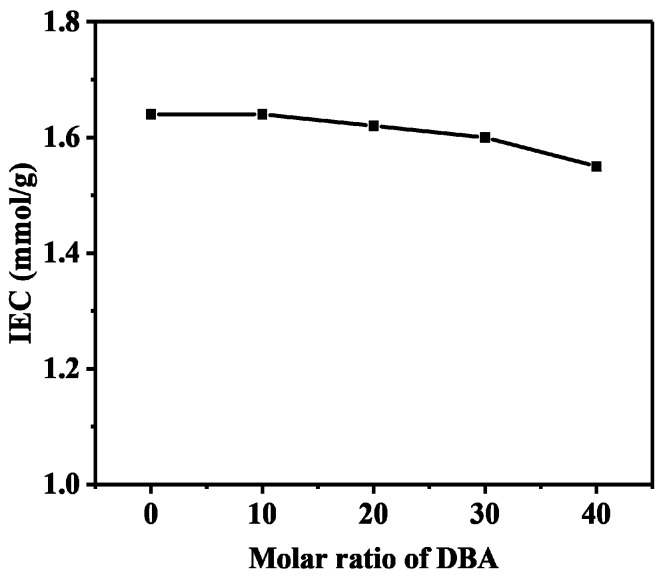
The ion exchange capacity (IEC) values of CSPEN-60-DBA-x.

The ion exchange capacity (IEC) represents the number of protons that can be replaced in proton exchange membranes, which play an important role in the water uptake, proton conductive, and swelling ratio of membranes. While proper water absorption facilitates the formation of a continuous hydrophilic channel for proton transport in PEMs, excessive absorption of water molecules reduces the mechanical properties and dimensional stability of PEMs. As shown in Figure 6, the IEC values of CSPEN-60-DBA-x membranes decrease from 1.64 to 1.55 mmol·g^−1^ with the increasing propenyl group contents. This is mainly due to the fact that the crosslinking network reduces the mobility of the sulfonic acid molecule and limits the replacement of H^+^ by Na^+^.

**Figure 7:**
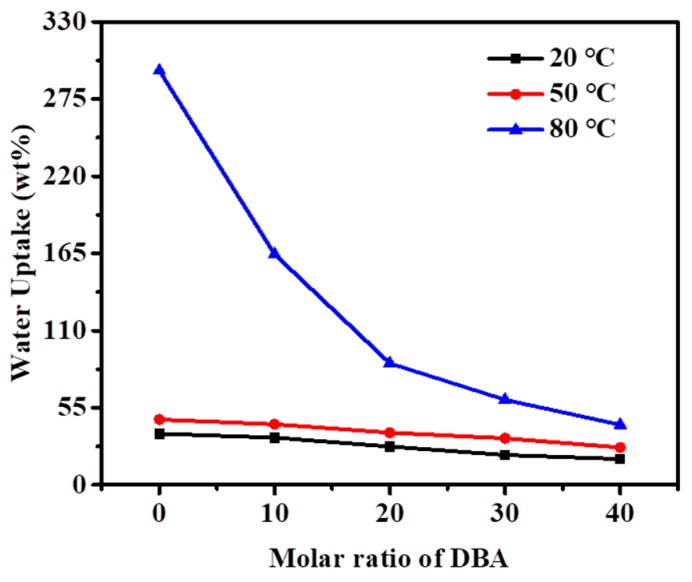
The water uptake of CSPEN-60-DBA-x at different temperatures.

In the proton transport mechanism, sulfonated polymer membranes utilize water molecules as proton carriers to transport protons. Therefore, it is particularly important to study the water uptake of polymer membranes in the fuel cell. As shown in Figure 7, the water uptake of CSPEN-60-DBA-x sulfonated poly(aryl ether nitrile) membranes with different propenyl group contents increase with the increasing temperature, while the water uptake of sulfonated poly(aryl ether nitrile) membranes decreases with the increasing contents of the propenyl group at the same temperature. For instance, the water uptake of SPEN-60-DBA-0 is 36.5 wt% at room temperature and water uptake of SPEN-60-DBA-0 is 295.2 wt% at 80 °C. In particular (at 80 °C), the water uptake of CSPEN-60-DBA-40 is only 42.9 wt%. This phenomenon is mainly due to the formation of the three-dimensional cross-linking network in sulfonated poly(aryl ether nitrile) at cross-linked temperature. The cross-linked network restricts the movement of sulfonated poly(aryl ether nitrile) molecular chains and makes the internal structure of CSPEN membranes tighter, thus reducing the free volume of sulfonated poly(aryl ether nitrile). As a result, the hydrophilic region is limited and the absorption of water molecules is reduced. Moreover, the network structure of CSPEN-60-DBA-x films becomes more compact with the increase of propenyl group contents.

**Figure 8:**
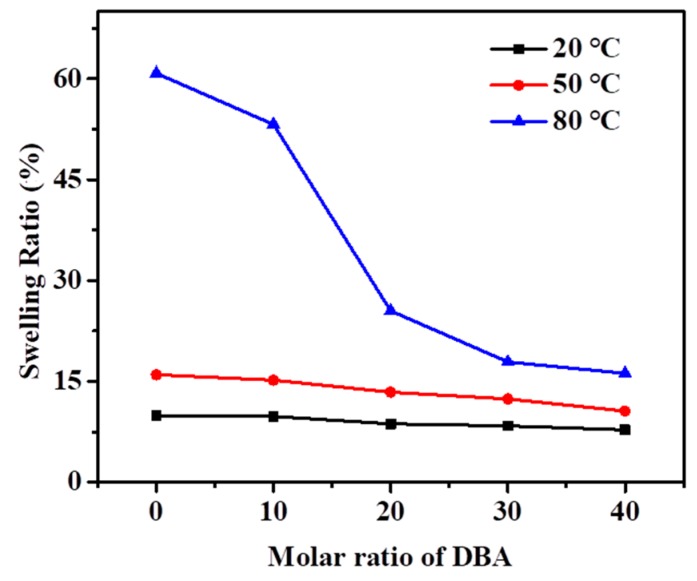
The swelling ratio of CSPEN-60-DBA-x at different temperatures.

Furthermore, it can be seen from Figure 8 that the swelling ratio of sulfonated poly (aryl ether nitrile) membranes shows a similar trend as that of the above-mentioned water uptake. The swelling ratio of sulfonated poly (aryl ether nitrile) membranes increases with temperature and decreases with the increase of propenyl group contents. For example, the swelling ratio of SPEN-60-DBA-0 membranes increased from 9.9% to 60.8% when the temperature was raised from 20 °C to 80 °C, and that of CSPEN-60-DBA-40 membranes was only 16.2% at 80 °C. This mainly results from the formation of the three-dimensional cross-linking network in sulfonated poly (aryl ether nitrile). This cross-linking network restricts the movement of the molecular chain of sulfonated poly (aryl ether nitrile) and makes the CSPEN-60-DBA-x membranes more compact, which effectively restricts the swelling of the CSPEN-60-DBA-x. It was probably attributed to the increasing interaction of the polymers resulting from cross-linking in the structures, which limited swell volume with respect to water uptake. These results indicate that crosslinking restricted the hydrophilic domains, thus decreased the water uptake and swelling ratio. As expected, all the cross-linked membranes exhibited excellent dimensional stabilities which increase with the increase of propenyl group contents.

### 3.6. Proton Conductivity

**Figure 9:**
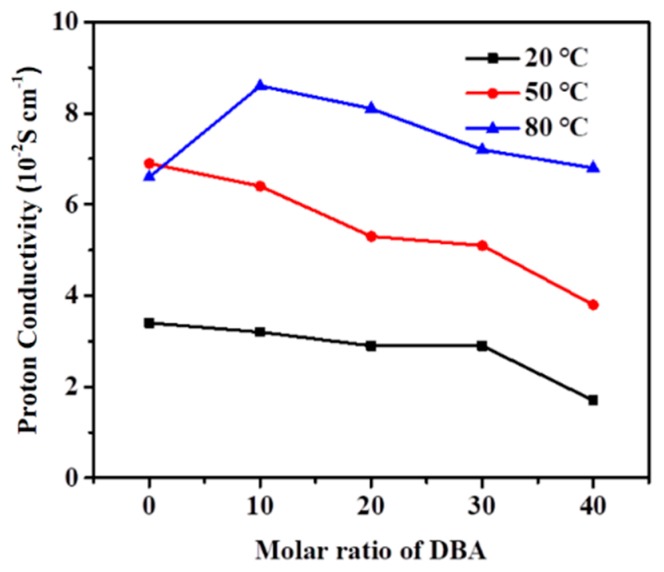
The proton conductivity of CSPEN-60-DBA-x at different temperatures.

In the proton exchange membrane fuel cell system, the proton conductivity of the membranes higher than 0.01 S·cm^−1^ is a prerequisite for evaluating the performance of the fuel cell. Figure 9 and Table 3 display the temperature dependent proton conductivity of CSPEN-60-DBA-x membranes (x refers to the propenyl group contents). As revealed in Figure 9, the proton conductivity of CSPEN-60-DBA-x membranes increases significantly with the increase of temperature, which due to the promoted the molecular mobility of water and polymer chains, thus enhancing the mobility of hydrated protons. Moreover, the proton conductivity of CSPEN-60-DBA-x membranes decreased with the increase of propenyl group contents, that is to say, it has the same trend as water uptake. This is attributed to the fact that the formation of cross-linking network reduces the movement of molecular segments and the free volume of the membrane, thus impeding the water absorption and proton mobility of the membranes. It is also found that the proton conductivity of SPEN-60-DBA-0 without cross-linking decreases at 80 °C while the proton conductivity of CSPEN-60-DBA-x membranes increases. It can be inferred that the excessive swelling of CSPEN membranes is inhibited by cross-linking at high temperature. Therefore, the sulfonic group content in per unit volume of CSPEN membranes is not too diluted and remained at a high level, hence the cross-linked membranes still maintain a high proton conductivity. Furthermore, the proton conductivity of commercial Nafion 117 membranes and previously reported Disulfonated-6F-PAE-CN (35)/FSPES-2 membranes in 100% RH at 20 °C is slightly higher than that of the cross-linked membranes, which is attributed to the large rigidity of CSPEN backbone and relatively weak proton carrying capacity. All the proton conductivity of CSPEN-60-DBA-x membranes higher than 0.01 S·cm^−1^, which meets the application requirements of the sulfonated poly (aryl ether nitrile) membranes as PEM materials in fuel cells.

### 3.7. Methanol Permeability and Selectivity

**Figure 10:**
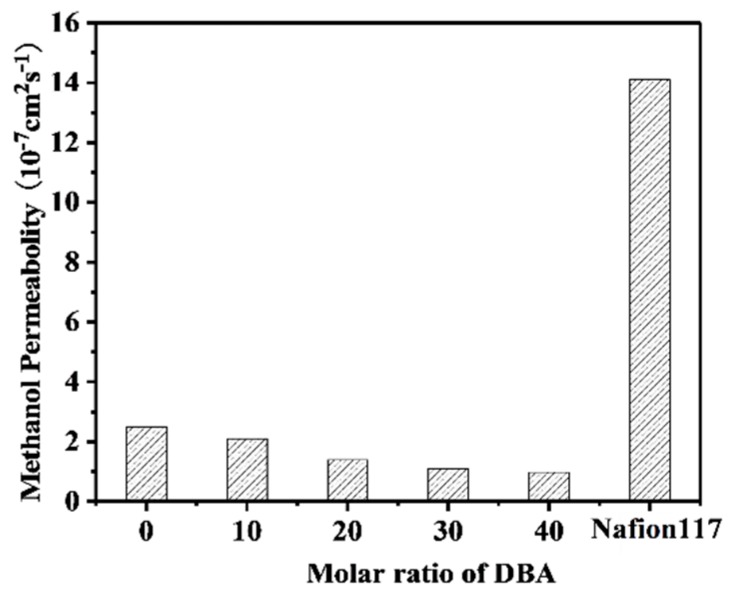
The methanol permeability of CSPEN-60-DBA-x at different temperatures.

Methanol permeability is one of the key factors to evaluate the performance of direct methanol fuel cells. The proton exchange membrane used in direct methanol fuel cells should possess good methanol resistance. As is shown in Figure 10 and Table 3, methanol permeability of all CSPEN-60-DBA-x is much lower than that of previously reported Disulfonated-6F-PAE-CN (35)/FSPES-2 membranes(8.7/8.0 × 10^−7^ cm^2^·s^−1^) and Nafion 117 (14.1 × 10^−7^ cm^2^·s^−1^) in 100% RH, and the numerical range is 0.98–2.5 × 10^−7^ cm^2^·s^−1^. In the proton exchange membranes, water and methanol use the same channel to pass through. After crosslinking by heat treatment, the propenyl group in the membranes effectively introduce a three-dimensional crosslinking network. The tight structure of the crosslinking network effectively limits the hydrophilic domain and inhibits the swelling of the membranes. At the same time, the formation of a cross-linking network narrows the transport channel of methanol, and plays a strong role as a barrier in methanol permeation.

**Figure 11:**
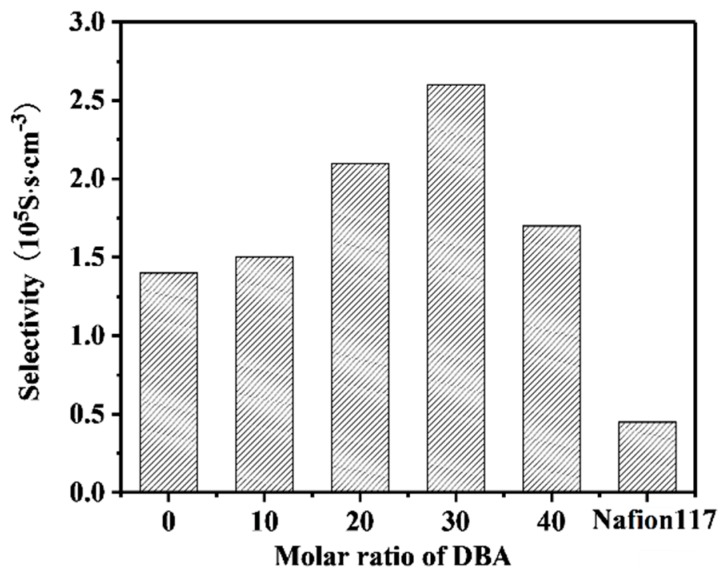
The selectivity of CSPEN-60-DBA-x at different temperatures.

Selectivity, calculated by the ratio of proton conductivity to methanol permeability, is an important parameter to evaluate the comprehensive performance of proton exchange membranes. It reflects the comprehensive ability of proton exchange membranes to conduct protons and methanol resistance. As is shown in Figure 11 and Table 3, the selectivity of all membranes is higher than that of Nafion 117 and previously reported Disulfonated-6F-PAE-CN (35)/FSPES-2 membranes. In addition, the selectivity of CSPEN-60-DBA-x increases at first and then decreases with the increasing of propenyl group contents. The methanol permeability of CSPEN-60-DBA-x polymer decreases much more than that of proton conductivity at the propenyl group contents less than 30%. When the content of the propenyl group exceeds 30%, the denser cross-linking structure leads to a more remarkable decrease in the proton conductivity of polymer membranes than that of methanol permeability. CSPEN-60-DBA-30 has the highest selectivity among all membranes, reaching 2.6 × 10^5^ S·s·cm^−3^, about 5.8 times that of Nafion 117.

## 4. Conclusions

Novel sulfonated poly(aryl ether nitrile)s have been synthesized through fixing the molar content of sulfonated group in bisphenol monomer at 60%, and tuning the content of the propenyl group in sulfonated poly(aryl ether nitrile)s. The cross-linking reaction of the propenyl groups in the SPENs is cured at 230 °C, and the sulfonic acid groups in these SPENs are stable under this treatment condition. All the results show that this method is an effective way to improve the dimensional stability and methanol permeability to meet the application requirements of the sulfonated poly (aryl ether nitrile) membranes as proton exchange membrane materials in fuel cells. For example, CSPEN-60-DBA-30 membranes shown the proton conductivity of 0.072 S·cm^−1^ at 80 °C, and the methanol permeability of all CSPEN-60-DBA-x is much lower than that of Nafion 117 (14.1 × 10^−7^ cm^2^·s^−1^), and the numerical range is 0.98–2.5 × 10^−7^ cm^2^·s^−1^. Especially, it is found that the selectivity of CSPEN-60-DBA-x increases at first, and then decreases with the increasing of propenyl group contents.

## Figures and Tables

**Table 1:** Raw material ratio for the synthesis of side chain crosslinkable sulfonated poly(aryl ether nitrile) containing allyl bisphenol A with different molar fractions.

Copolymers	Allyl bisphenol A (mol)	Bisphenol A (mol)	SHQ (mol)	K_2_CO_3_ (mol)	DFBN (mol)	NMP (mL)	Toluene (mL)
SPEN-60-DBA-0	0.00	0.04	0.06	0.2	0.1	65	25
CSPEN-60-DBA-10	0.01	0.03	0.06	0.2	0.1	65	25
CSPEN-60-DBA-20	0.02	0.02	0.06	0.2	0.1	65	25
CSPEN-60-DBA-30	0.03	0.01	0.06	0.2	0.1	65	25
CSPEN-60-DBA-40	0.04	0.00	0.06	0.2	0.1	65	25

**Table 2:** The gel content of CSPEN-60-DBA-x with different propenyl group contents.

Membranes	SPEN-60-DBA-0	CSPEN-60-DBA-10	CSPEN-60-DBA-20	CSPEN-60-DBA-30	CSPEN-60-DBA-40
The gel content (%)	0	14	26	43	58

**Table 3:** The proton conductivity, methanol permeability, and selectivity of CSPEN-60-DBA-x at different temperatures.

Membranes	Proton conductivity (10^−2^ S·cm^−1^)			Methanol permeability (10^−7^ cm^2^·s^−1^)	Selectivity (10^5^ S·s·cm^−3^)
	20 °C	50 °C	80 °C		
SPEN-60-DBA-0	3.4	6.9	6.6	2.5	1.4
CSPEN-60-DBA-10	3.2	6.4	8.6	2.1	1.5
CSPEN-60-DBA-20	2.9	5.3	8.1	1.4	2.1
CSPEN-60-DBA-30	2.9	5.1	7.2	1.1	2.6
CSPEN-60-DBA-40	1.7	3.8	6.8	0.98	1.7
Nafion117	6.4	--	--	14.1	0.45
Disulfonated-6F-PAE-CN (35) [35]	6.5	--	--	8.7	0.75
FSPES-2 [51]	7.4	--	--	8.0	0.93
